# Factors Associated with Increased Alpha-Tocopherol Content in Milk in Response to Maternal Supplementation with 800 IU of Vitamin E

**DOI:** 10.3390/nu11040900

**Published:** 2019-04-22

**Authors:** Amanda de Sousa Rebouças, Ana Gabriella Costa Lemos da Silva, Amanda Freitas de Oliveira, Lorena Thalia Pereira da Silva, Vanessa de Freitas Felgueiras, Marina Sampaio Cruz, Vivian Nogueira Silbiger, Karla Danielly da Silva Ribeiro, Roberto Dimenstein

**Affiliations:** 1 Department of Biochemistry, Federal University of Rio Grande do Norte, 59078-970 Natal-RN, Brazil; amandasousar2@hotmail.com (A.d.S.R.); rdimenstein@gmail.com (R.D.); 2 Department of Nutrition, Federal University of Rio Grande do Norte, 59078-970 Natal-RN, Brazil; gabriella_lemos_06@yahoo.com.br (A.G.C.L.d.S.); amandda_freitas@outlook.com (A.F.d.O.); lorenathaliaps@gmail.com (L.T.P.d.S.); vanessadffelgueiras@gmail.com (V.d.F.F.); 3 Department of Pharmacy, Federal University of Rio Grande do Norte, 59012-570 Natal-RN, Brazil; marinasmcruz@gmail.com (M.S.C.); viviansilbiger@hotmail.com (V.N.S.)

**Keywords:** clinical trial, lactation, infants, breastfeeding, lactating women

## Abstract

Background: Vitamin E supplementation might represent an efficient strategy to increase the vitamin E content in milk. The present study aimed to evaluate the impact of supplementation with 800 IU RRR-alpha-tocopherol on the alpha-tocopherol content of milk and the factors associated with the increase in vitamin E. Methods: Randomized clinical trial with 79 lactating women from Brazil, who were assigned to the control group, or to the supplemented group (800 IU of RRR-alpha-tocopherol). Milk and serum were collected between 30 and 90 days after delivery (collection 1), and on the next day (collection 2). Alpha-tocopherol was analyzed using high-performance liquid chromatography. Results: In the supplemented group, the alpha-tocopherol content in serum and milk increased after supplementation (*p* < 0.001). In the multivariate analysis, only alpha-tocopherol in milk (collection 1) was associated with the level of this vitamin in milk after supplementation (*β* = 0.927, *p* < 0.001), and binary logistic regression showed that the dietary intake was the only determinant for the greater effect of supplementation in milk. Conclusion: The pre-existing vitamin level in milk and diet are determinants for the efficacy of supplementation in milk, suggesting that in populations with vitamin E deficiency, high-dose supplementation can be used to restore its level in milk.

## 1. Introduction

Breast milk contains all the essential nutrients and factors for the growth and development of the infant’s gastrointestinal, cerebral and immune system [1,2]. Thus, exclusive breastfeeding is recommended during the first six months of life [3]. Among the vitamins present in milk, vitamin E, is an antioxidant responsible for protecting the lipoproteins and polyunsaturated fatty acids present in the cellular membranes against peroxidation [4]. Vitamin E deficiency in children and newborns, including preterm infants (birth <37 gestational weeks), can lead to intracranial hemorrhage, chronic pulmonary diseases, hemolytic anemia, retinopathy and childhood cognitive deficits [4]. The prevalence of vitamin E deficiency (SVD) in newborns can be up to 77% [5,6,7] and in Brazil, a study found low vitamin levels (<500 μg/dL) in 90% of newborns [8]. The transfer of vitamin E to breast milk depends on circulating lipoproteins, and this mechanism can be influenced by maternal factors, both intrinsic and extrinsic [2,9,10]. In colostrum milk, the actions of pregnancy hormones, such as estrogen, contributes to the increase in circulating lipoproteins, ensuring a greater transfer of vitamin E into milk [2,11]. However, in mature milk the vitamin E content decreases because of changes in fat globules, and other characteristics, such as maternal age, gestational age of delivery, and the fatty acid profile might that influence the vitamin E content in milk [8,9,12,13].

Studies analyzing this micronutrient in mature milk observed that even in lactating women with vitamin E deficiency, its concentration in milk was maintained, which suggested a possible mobilization of alpha-tocopherol from the adipose tissue, which is considered the largest extrahepatic vitamin E reserve [2,8,14,15].

One strategy to increase the concentrations of vitamin E in milk is maternal supplementation [9,10]. Garcia et al. [16] found that at 24 h after supplementation, alpha-tocopherol levels in the colostrum increased. Other studies [17,18] found that vitamin E supplementation in its naturally occurring form (RRR-alpha-tocopherol) is more efficient to increase its content in milk compared with supplementation with the synthetic form or with a blend of natural and synthetic forms. In the natural form, the lateral chain has the RRR conformation, whereas the synthetic form can present isomers with 2R- (RRR-, RSR-, RSS- and RRS-) and 2S- (SRR-, SRS-, SSR-, SSS-) conformations. This structural difference results in increased bioavailability of the RRR form because of its higher affinity for the liver alpha-tocopherol transfer protein (alpha-TPP) [18,19].

Single-dose supplementation with 400 IU RRR-alpha-tocopherol in the immediate postpartum period caused an increase in the vitamin in the transitional milk (between 7 and 15 days after delivery), but not in the mature milk [20,21]. The authors suggested that a higher dose of vitamin E could influence the duration of the response. This identified the need to investigate the effect of higher doses, because the studies only used 400 IU of alpha-tocopherol, and suggested that this supplementation should be provided in the mature milk phase, which comprises a period of greater stability in milk nutritional composition.

Interestingly, different responses to supplementation have been noted, where the same treatment caused a greater increase of the vitamin in the milk in some studies [20,21,22] and a smaller effect in others [17], however, these studies lacked an analysis of the factors that influenced this response. These observations should be considered, because maternal milk with a low alpha-tocopherol content has been found, which could expose infants to vitamin E deficiency (VED) [20,21,23,24,25]. By contrast, studies of vitamin E supplementation in a single dose and in greater quantity could reveal the previously unknown mechanism of vitamin transfer to the mammary gland.

Thus, given that maternal supplementation with vitamin E is an effective measure to increase this vitamin content in milk [21,22], the mother-child binomial should be protected from the adverse effects of VED, and that there are differences in the response to this supplementation, but no understanding of which characteristics contribute to this response. The objective of the present study was to evaluate the impact of supplementation with 800 IU RRR-alpha-tocopherol on the alpha-tocopherol level in mature milk and the factors associated with the increase, with the aim of improving our understanding of the mechanism the transfer of vitamin E in the lactation period.

## 2. Materials and Methods

### 2.1. Participants and Intervention

The study was a randomized, parallel-group trial. Participants were recruited at the Pediatric Ambulatory Care of the Onofre Lopes University Hospital (HUOL), Natal-RN, Brazil, and data collection took place between October 2017 and July 2018.

The present study was approved by the Ethics Committee of the Federal University of Rio Grande do Norte (UFRN), under the protocol number 2.327.614, CAAE 76779217.1.0000.5537, and was also registered in the Brazilian Registry of Clinical Trials—ReBec, under the code RBR-38nfg2, available at http://www.ensaiosclinicos.gov.br/rg/RBR-38nfg2/.

The sample calculation was performed using GPower software, Version 3.1.9 [26] considering two independent groups tested using one way analysis of variance (ANOVA) for repeated measures among factors, with alpha parameters equal to 5%, expected power at 80%, and the effect measure value equal to 0.25 [27]. The analysis showed that each group should have at least 33 individuals, totaling 66 participants.

The eligibility criteria included women between 30 and 90 days after delivery; who were breastfeeding their children, either exclusively or partially; who were residents of Natal, RN and its metropolitan regions; who were not diagnosed with a diseases (hypertension, diabetes, neoplasms, heart disease, diseases of the gastrointestinal and hepatic tract, syphilis or were HIV-positive); who were non-smokers; no multiple births and whose infants were not malformed. Exclusion criteria were women who did not have sufficient milk or blood for analysis of vitamin levels, users of illicit drugs, and those who made daily use of vitamin supplements containing vitamin E during lactation.

**Figure 1:**
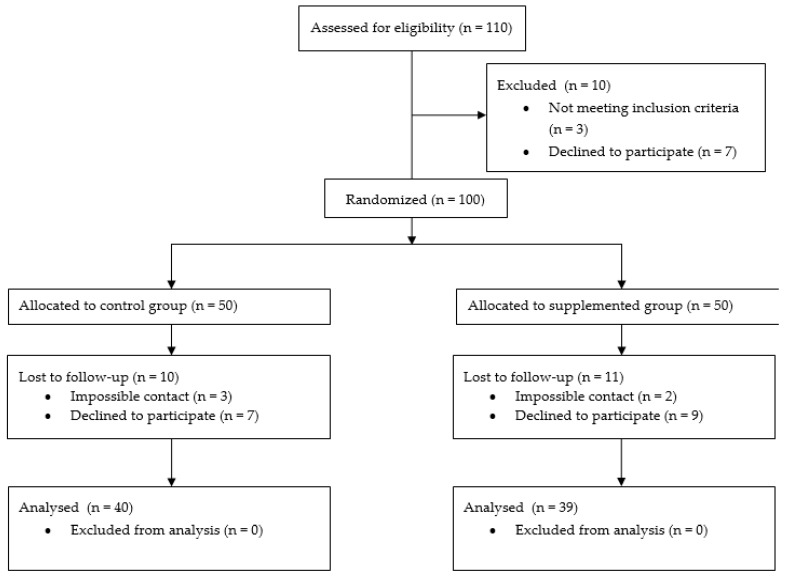
Consolidated Standards of Reporting Trials flow diagram (CONSORT).

The eligible participants were informed of the study’s objectives and those who agreed to participate signed the consent form. At recruitment, they were allocated in one of the study groups, depending on the day of the week: Monday and Thursday for the supplemented group and Tuesday and Wednesday for the control group, where only the supplemented group ingested two capsules containing 400 IU of RRR-alpha-tocopherol consecutively, totaling 800 IU (588 mg of alpha-tocopherol). The capsule contained 98% RRR-alpha-tocopherol acetate, as assessed according to the method of Lira (2017) [21]. The study complied with the Consolidated Standards of Reporting Trials—CONSORT (Figure 1).

### 2.2. Data Collection

A semi-structured questionnaire was used to collect data on socioeconomic aspects, such as family income, schooling and maternal age, as well as information on gestational age and type of delivery. Maternal height and current weight were also assessed and used to calculate the body mass index (BMI).

Milk and serum were collected from the participants at two time points. Collection 1 was performed at the hospital and collection 2 was performed at the participant’s home the day after collection 1. In the supplemented group, supplementation with 800 IU of RRR-alpha-tocopherol was performed immediately after collection 1 of milk and serum.

A 2 mL sample of breast milk was collected by manual expression from a single breast that had not breastfed recently, and 5 mL of blood were collected by venipuncture. All the biological samples were collected after a 4 to 6 h fast, stored in polypropylene tubes packed in aluminum foil, and transported in refrigerated units. The breast milk was stored at −20 °C until the time of analysis. Before storage, the blood samples were centrifuged for 10 min (at 4000 rpm), to separate the serum for analysis of vitamin E and lipoproteins.

The dietary intake of vitamin E was evaluated by means of the 24 h dietary recall (24HR) applied at the two collection time points. Participants were asked about all foods, supplements and beverages consumed the day before the interview.

### 2.3. Determination of Alpha-Tocopherol and Lipid Profile in Biological Samples

The extraction of alpha-tocopherol from milk and serum was performed according to the method adapted by Lira et al. (2013) [28]. Ethanol (95%) was used to precipitate proteins (Vetec, Rio de Janeiro, Brazil), and hexane PA (Vetec, Rio de Janeiro, Brazil) was used as an extraction reagent. After evaporation in nitrogen, serum and milk residues were dissolved, respectively, in 250 μL of absolute ethanol (Vetec, Rio de Janeiro, Brazil) and 250 mL of dichloromethane (Vetec, Rio de Janeiro, Brazil): methanol (Sigma-Aldrich, St. Louis, Missouri, EUA) (2:1; *v*/*v*). The aliquots were then analyzed using high-performance liquid chromatography (HPLC).

HPLC consisted of an LC-20AT (Shimadzu, Kyoto, Japan) pump coupled to a CBM 20A communicator and an SPD-10A UV-VIS detector (Shimadzu, Kyoto, Japan). AC18 reversed phase column (LiChroCART 250-4, Merck, Darmstadt, Germany) was used for chromatographic separation. The mobile phase was 100% methanol in an isocratic system, with a flow rate of 1 mL/min and a wavelength of 292 nm was used to detect alpha-tocopherol. The identification and quantification of the vitamin in the samples were established by comparing the area of the peak obtained in the chromatogram with the area of the alpha-tocopherol standard (Sigma-Aldrich, São Paulo, Brazil). The concentration of the standard was confirmed by the specific extinction coefficient for alpha-tocopherol (e1%, alpha-tocopherol, 1 cm = 75.8 to 292 nm) in absolute ethanol (Vetec, Rio de Janeiro, Brazil) [29]. Women with serum alpha-tocopherol values less than 12 μmol/L were considered as deficient in vitamin E [30].

The transport of vitamin E from serum to breast milk involves lipoproteins; therefore, serum cholesterol and high-density lipoprotein (HDL) levels were analyzed with using a commercial kit (Labtest) and enzymatic colorimetric methods, by using an automatic biochemistry analyzer (Labmax plenno). Low-density lipoprotein (LDL) was quantified using the equation proposed by Martin et al. (2013) [31].

### 2.4. Dietary Intake of Vitamin E

Vitamin E intake was obtained using two 24 h dietary recall (24HR), applied using face-to-face interviews at the two data collection times in both groups. During this interview, the participants were asked about the food (and its preparation), supplements and beverages consumed in the last 24 h before the interview, in which the home measures described were converted to grams or milliliters [32,33] and the amount of vitamin E consumed was analyzed using the software Virtual Nutri Plus [34], from the database constructed by Rodrigues (2016) [8]. The dietary intake of vitamin E was corrected for total energy intake. The resulting values were obtained using SPSS, version 21.0 for Windows (SPSS Inc., Chicago, IL, USA) employing the residual method.

### 2.5. Statistical Analysis

Statistical analysis was performed using the statistical software IBM SPSS version 21.0 for Windows (SPSS Inc., Chicago, IL, USA). The Kolmogorov–Smirnov normality test was applied. Numerical data were expressed as the mean (standard deviation, SD), and categorical results were reported as absolute and relative frequencies. Student’s *t*-test for dependent samples was used to verify intragroup differences, and the *t*-test for independent samples was used to analyze the differences between the groups. To evaluate the relationship between serum, breast milk and dietary vitamin E intake, the Pearson correlation coefficient was calculated. Linear multiple regression analysis was used to verify the ratio between alpha-tocopherol in milk after vitamin supplementation and in serum, the lipid profile, vitamin E intake and other maternal factors. The factors associated with the effect of supplementation on milk were also investigated. For this, the lactating women in the supplemented group were divided into quartiles according to the percentage increase in the milk alpha-tocopherol content between collection 1 and collection 2, being classified into a smaller effect (quartile 1) and greater effect (quartiles 2–4). The quartile categorization was used to identify the participants who presented lower effect and greater effects, because all participants should present higher alpha-tocopherol in milk values after supplementation. In addition to providing an analysis of the possible determinants for the milk supplementation response. The association of maternal variables with the effect of supplementation was evaluated according to binary multiple regression. All differences were considered significant when *p* ≤ 0.05.

## 3. Results

### 3.1. General Characteristics of the Population

The socioeconomic characteristics of the lactating women are presented in Table 1. The mean age of the participants was 27 years, and the majority had completed high school. About 40% of the women were overweight according to their BMI values, and exclusive breastfeeding was predominant (>84%) in both groups. The dietary intake of vitamin E was equivalent to 8.7 mg/day in the control, which was below the recommended intake (16 mg/day) [30] and there was no difference between the groups in terms of dietary intake of vitamin E (*p* = 0.901).

### 3.2. Effect of Vitamin E Supplementation on Serum and Breast Milk

At collection 1, the maternal serum alpha-tocopherol concentrations were similar between the control and supplemented groups, at 26.37 (4.6) μmol/L and 26.38 (5.4) μmol/L, respectively (*p* = 0.996). In the control group, there was no difference in the alpha-tocopherol concentrations between collection 1 and collection 2 (*p* > 0.05). Neither group contained cases of VED (<12 μmol/L). In addition, the lipid profiles were similar between the collections and between the groups (*p* > 0.05) (Table 2).

After supplementation with 800 IU RRR-alpha-tocopherol, a 183% increase in serum alpha-tocopherol was observed in the supplemented group (collection 2), reaching 48.27 μmol/L (*p* < 0.001) (Table 2).

For the alpha-tocopherol content in mature milk, the control group presented 6.91 (1.81) μmol/L and the supplemented group presented 6.98 (2.18) μmol/L (*p* = 0.883). One day after supplementation (collection 2), milk from the supplemented group presented higher levels of alpha-tocopherol (15 μmol/L) compared with that in the control group (6.94 μmol/L) (*p* < 0.001), an increase equivalent to 124% in the post-supplementation milk.

### 3.3. Factors Associated with Alpha-Tocopherol in Breast Milk after Supplementation

**Figure 2:**
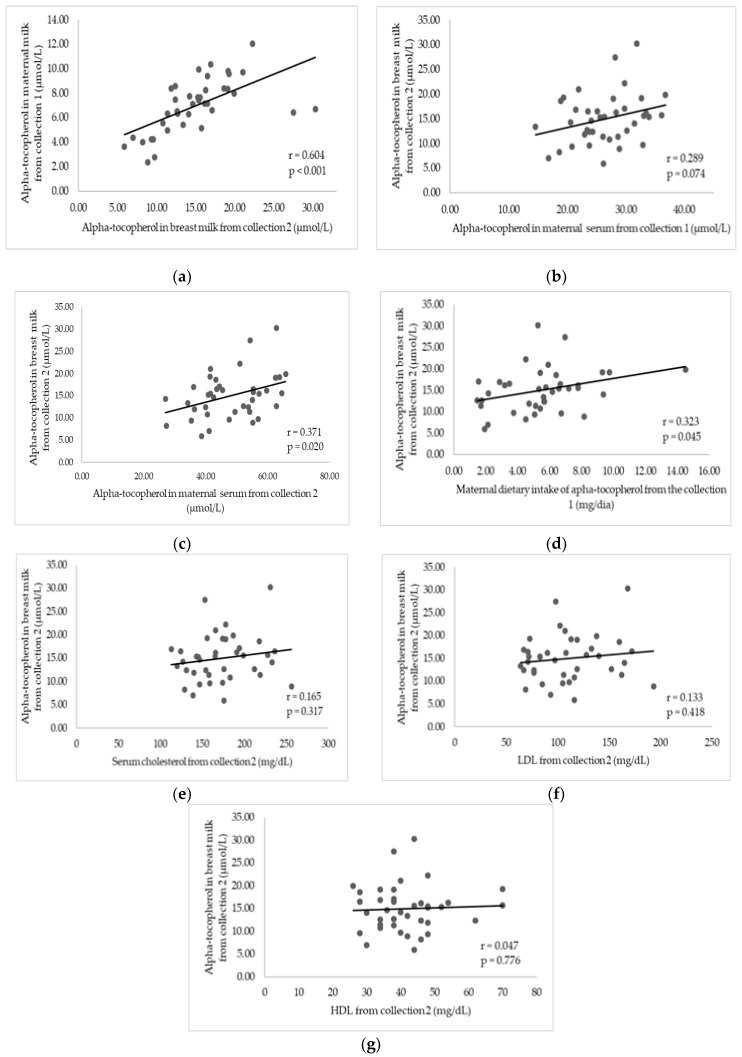
Correlations between alpha-tocopherol in breast milk obtained in collection 2 and maternal variables of the supplemented group. Natal, Rio Grande do Norte, Brazil, 2017–2018. (**a**) Correlation between alpha-tocopherol in breast milk from collection 1 and 2; (**b**) correlation between alpha-tocopherol in breast milk from collection 2 and serum from collection 1; (**c**) correlation between alpha-tocopherol in breast milk in serum from collection 2; (**d**) correlation between alpha-tocopherol in breast milk of collection 2 and the vitamin E intake of collection 1; (**e**) correlation between alpha-tocopherol in breast milk from collection 2 and serum cholesterol from collection 2; (**f**) correlation between alpha-tocopherol in breast milk from collection 2 and low-density lipoprotein (LDL) from collection 2; (**g**) correlation between alpha-tocopherol in breast milk from collection 2 and high-density lipoprotein (HDL) from collection 2. r = Pearson’s correlation coefficient; *p* value = level of significance (*p* < 0.05 = statistically significant).

In the supplemented group, after Pearson correlation analysis, milk from collection 1, dietary intake of vitamin E, and alpha-tocopherol in serum from collection 2 were identified as positively related to alpha-tocopherol levels in milk from collection 2 (Figure 2). These variables were included in the multiple linear regression analysis to evaluate the factors associated with the alpha-tocopherol concentration in breast milk after supplementation. Only alpha-tocopherol in the milk before supplementation was a determinant for the increase in the vitamin content in the milk after administration of 800 IU alpha-tocopherol (β = 0.927, *p* < 0.001, 95% CI 1.925–2.396). Thus, the higher the vitamin concentration in milk, the greater the transfer of the vitamin to the mammary gland.

When dividing the participants of the supplemented group according to the effect of supplementation (quartile 1 and quartiles 2–4), where quartile 1 is equivalent to 83% of the vitamin E increase percentage in milk after supplementation, we observed that the dietary intake of vitamin E was a determinant that caused a greater response to supplementation (*p* = 0.020, 95% CI 0.209–0.877), which suggested that the higher the intake, the greater the effect of supplementation (Table 3). The characteristics of the participants divided by the effect of supplementation are described in Table 4, which showed that the consumption of calories, alpha-tocopherol and total fat was higher in the group showing a higher effect of supplementation (*p* = 0.001, *p* = 0.013, *p* = 0.033, respectively).

## 4. Discussion

Mature milk is the most stable stage of lactation, in which the content of alpha-tocopherol is not influenced by pregnancy-related factors, as occurs in the colostrum [2,7]. It should be emphasized that the mature milk presents a higher concentration of lipids and in contrast, there is a lower secretion of alpha-tocopherol, suggesting that there are distinct mechanisms involved in the transfer of this vitamin into breast milk [2,35,36].

In the present study, the lactating women had adequate vitamin E status, in accordance with other studies considering the same stage of lactation [8,21]. However, a low dietary intake of vitamin E was noted (Table 1), which could trigger the mobilization of alpha-tocopherol from maternal reserves, such as the adipose tissue, into breast milk [15,19]. This low consumption of vitamin E was also reported in other populations in Brazil, Greece and Poland [21,25,36], which suggests a frequent inadequacy in vitamin E consumption during lactation.

Even in situations of inadequate consumption, the vitamin E concentration in milk was not influenced by diet and circulating maternal levels [8,20,21,37,38,39,40]. However, this present study was the first to identify a positive association between alpha-tocopherol levels in milk and ingested vitamin E (diet + supplementation) and with serum alpha-tocopherol (Figure 2d,c). This suggested that in high-consumption situations, the ingested and circulating maternal levels are the main factors responsible for the vitamin level in milk. It is likely that in situations of low vitamin E consumption (as found in the studies cited), milk vitamin E might originate from other sources, such as the body’s reserve [4], explaining the absence of a relationship between those variables.

To prevent of VED in infants, it is necessary for breast milk to contain adequate levels of vitamin E, so that children can obtain the benefits of the micronutrient, through the creation of vitamin reserves in the body and its antioxidant action [4,19]. Some studies that evaluated this vitamin in mature milk observed values below the nutritional requirements of infants [20,23,36,37], which suggested that maternal vitamin E supplementation could be an important strategy to increase milk vitamin contents [17,20,21].

In this study, supplementation with 800 IU of alpha-tocopherol caused a 183% increase in serum alpha-tocopherol, and a 124% increase in breast milk alpha-tocopherol (Table 2). Other clinical trials using a lower dose (400 IU alpha-tocopherol) found an increase of 60% to 80% in the vitamin content in milk after supplementation [18,20,21,22], showing a reduced effect compared with that shown in the present study. These findings suggested that the response to supplementation might be influenced by both the dosage used and the determinant factors. However, trials have not evaluated the factors associated with the different responses to large vitamin E doses [17,20], being important to understand how this vitamin is transferred into the mammary gland.

When analyzing the factors associated with a better response in milk after supplementation, it is important to highlight that only the content of this vitamin in the basal milk (before supplementation) and the dietary intake were demonstrated to increase the levels of this micronutrient in milk (Table 3), which suggested that the higher the consumption of vitamin E and its levels in milk, the greater the transfer of alpha-tocopherol from the supplement to the mammary gland.

Assessment of the profile of lactating women in the supplemented group, showed that the participants with the highest supplementation effect (quartiles 2–4) had a higher intake of calories, alpha-tocopherol and total fat (Table 4). These findings suggested that the amount of fat available in the diet may improve the bioavailability of the vitamin in the body, such as its absorption and distribution to tissues, and in this case, to the mammary gland, as reported in [41,42,43].

Such evidence also demonstrated that in mature milk, the Michaelis–Menten kinetic theory could not be applied, as it proposes that the transfer of vitamin to milk occurs through active transport, characterized by a saturation of the lipoprotein receptors in the breast tissue in situations of large contents of vitamin E, which would prevent the continuous transfer of the vitamin into the breast milk after supplementation [38,44]. Notably, in a study of dairy cows, Weiss and Wyatt (2003) [45] suggested that the ability of lipoproteins to carry alpha-tocopherol could determine the uptake of this vitamin by the breast tissue, and that the limiting factor for this mechanism would be the maximum content of vitamin E in the lipoproteins.

To further investigate this relationship between lipoproteins and vitamin E transport, we determined the circulating lipoproteins and the serum cholesterol and triglycerides profiles; however, no relation between them and the response to supplementation was found (Table 2). In fact, the mechanism of transport of vitamin E to the mammary gland is poorly understood [2,46,47]. Circulating lipoproteins are responsible for this transfer, with LDL being the main carrier [44,48]; however, transport may occur in the presence or absence of its receptors in the mammary gland [2,44]. Other receptors are found in breast tissue, such as Scavenger Receptor B-1 (SR-B1), which has binding sites for both LDL and HDL, and CD36, which has high affinity binding sites for HDL, LDL, and very low-density lipoprotein (VLDL) [2,48]. It has also been suggested the participation of lipoprotein lipase (LPL), which may show increased activity during lactation, contributes to the greater circulation of alpha-tocopherol and its uptake [49].

These findings provide important information to understand the mechanisms by which vitamin E is transferred into the mammary gland, demonstrating that, in situations of supplementation with 800 IU of vitamin E and its effect in mature milk, the better the vitamin E status (considering the milk and dietary intake), the more effective uptake into the mammary gland will be, regardless of receptor saturation. Further investigation into how this transport occurs is required, by means of in vitro and in vivo studies and using labeled isotopes of alpha-tocopherol, for example, to investigate its biotransformation. Notably, in populations with dietary inadequacy and low contents of vitamin E in milk, supplementation with higher doses of the vitamin, such as 800 IU alpha-tocopherol, might be required to obtain a more effective intervention. The analysis of a single dose during the day allowed us to investigate possible factors that could interfere with the response to supplementation; however, it is necessary to analyze how long the effect of this supplementation could be sustained, its contribution to maternal and infant nutritional status, and the use of smaller daily doses.

Therefore, supplementation associated with an adequate intake of vitamin E is an effective strategy to increase vitamin E levels in breast milk and prevent cases of vitamin E deficiency in infants, especially premature infants [2,4,11].

## 5. Conclusions

Vitamin E supplementation increased vitamin levels in milk and in maternal serum, and a positive relationship was found between alpha-tocopherol levels in milk, serum and dietary intake of vitamin E. Factors associated with the increase in alpha-tocopherol contents in milk after maternal supplementation with 800 IU of vitamin E were the basal levels of alpha-tocopherol in milk and the dietary intake of vitamin E.

## Figures and Tables

**Table 1:** Characterization of the 79 lactating women randomized into the control and supplemented groups of the study. Natal, Rio Grande do Norte, Brazil, 2017–2018.

Characteristics	Control Group *n* = 40	Supplemented Group *n* = 39	*p*-Value
Maternal age (years), mean (SD)	27 (6.8)	27 (6.8)	0.833 *
Postpartum age (days), mean (SD)	57 (25.8)	56 (23.7)	0.833 *
Education level *n*, (%)			
Incomplete primary education	4 (10.0)	5 (12.8)	0.149
Complete primary education	3 (7.5)	2 (5.1)	
Incomplete secondary education	14 (35.0)	6 (15.4)	
Complete secondary education	16 (40.0)	22 (56.4)	
Complete higher education	3 (7.5)	4 (10.3)	
Family income level *n*, (%) ^a^			
<1 Minimum wage	16 (40.0)	23 (59.0)	0.092
>1 Minimum wage	24 (60.0)	16 (41.0)	
Type of delivery *n*, (%)			
Vaginal	15 (37.5)	13 (33.3)	0.699
Caesarian	25 (62.5)	26 (66.7)	
Parity status *n*, (%)			
Primiparous	21 (52.5)	17 (43.6)	0.405
Multtiparou	19 (47.5)	22 (56.4)	
BMI classification (kg/m^2^), (%) ^b^			
Low weight	1 (2.5)	0 (0)	0.735
Normal	15 (37.5)	18 (46.2)	
Overweight	16 (40.0)	13 (33.3)	
Obese	8 (20.0)	8 (20.5)	
Type of maternal breastfeeding *n*, (%)			
Exclusive maternal breastfeeding	35 (87.5)	33 (84.6)	0.711
Maternal breast milk and other milks	5 (12.5)	6 (15.4)	
Calorie intake (Kcal/day), mean (SD)	3248.4 (711.2)	3270.7 (868.4)	0.970 *
Intake of alpha-tocopherol (mg/day), mean (SD)	8.7 (3.4)	8.8 (3.5)	0.901 *
Intake of total fat (g/dia), mean (SD)	69.2 (23.6)	69.9 (25.8)	0.905 *

*n*: number; BMI: Body Mass Index; SD: Standard deviation; Chi-square test. * Student’s *t*-test for independent samples used for the variables maternal age, postpartum age, calorie consumption, alpha-tocopherol and total fat; ^a^ Brazilian minimum wage per month = US$ 291.5; ^b^ WHO classification, 2000. *p*-value = level of significance (*p* < 0.05 = statistically significant).

**Table 2:** Maternal biochemical indicators of the control and supplemented groups in collections 1 and 2, performed in the study. Natal, Rio Grande do Norte, Brazil, 2017–2018.

Biochemical Indicators Evaluated	Control Group				Supplemented Group				Differences between Control Group and Supplemented Group	
	Collection 1	Collection 2	Change	*p*-Value *	Collection 1	Collection 2	Change	*p*-Value *	*p*-Value **	
									Collection 1 CG × SG	Collection 2 CG × SG
**Serum alpha-tocopherol (µmol/L)**	26.37 (4.6)	26.34 (4.92)	0.03 (2.5)	0.876	26.38 (5.4)	48.27 (10.5)	21.89 (7.4)	0.001	0.996	<0.001
**Alpha-tocopherol in breast milk (µmol/L)**	6.91 (1.8)	6.94 (2.0)	0.03 (1.2)	0.935	6.98 (2.2)	15.00 (5.1)	8.02 (4.2)	<0.001	0.883	<0.001
**Serum cholesterol (mg/dL)**	177 (41.0)	178 (42.0)	1.70 (33.5)	0.750	179 (44.0)	173 (36.0)	6.28 (16.0)	0.190	0.834	0.498
**Serum triglycerides (mg/dL)**	143 (99.0)	130 (86.0)	13.38 (57.8)	0.151	129 (66.0)	109 (51.0)	19.23 (32.9)	0.08	0.439	0.195
**HDL (mg/dL)**	40 (14.0)	41 (15.0)	0.25 (12.9)	0.903	42 (11.0)	42 (10.0)	0.31 (8.0)	0.811	0.574	0.724
**LDL (mg/dL)**	111 (35.0)	114 (40.0)	3.28 (34.4)	0.535	113 (43.0)	110 (35.0)	3.74 (14.7)	0.129	0.762	0.603

HDL: High-density lipoprotein; LDL: Low-density lipoprotein; ( ) Standard deviation; * Student’s *t*-test for dependent samples; ** Student’s *t*-test for independent samples. CG: Control group; SG: Supplemented group.

**Table 3:** Binary logistic regression model for variables associated with greater effect of 800 IU alpha-tocopherol supplementation in breast milk in the supplemented group.

Variables	Greater Effect of Supplementation (Quartiles 2–4) *	
	95% CI	*p*-Value
Alpha-tocopherol in milk collection 1 (µmol/L)	0.998–1.024	0.104
Alpha-tocopherol in serum collection 1 (µmol/L)	0.991–1.005	0.565
Alpha-tocopherol in serum collection 2 (µmol/L)	0.995–1.002	0.387
Dietary intake of vitamin E collection 1 (mg/day)	0.209–0.877	0.020 **
Serum cholesterol collection 1 (mg/dL)	0.937–1.163	0.431
LDL collection 1 (mg/dL)	0.846–1.051	0.289

* Above 83% of the vitamin E increase percentage in milk after supplementation. LDL: Low-density lipoprotein. *p*-value = level of significance. ** Significant difference.

**Table 4:** Characterization of the 39 lactating women randomized into the supplemented group, divided by the effect of milk supplementation (lower effect: quartile 1, equivalent to 83% of the vitamin increase percentage in the milk, and greater effect: quartiles 2–4). Natal, Rio Grande do Norte, Brazil, 2017–2018.

Characteristics	Quartile 1 *n* = 9	Quartiles 2–4 *n* = 30	*p*-Value
Maternal age (years), mean (SD)	27 (6.5)	27 (6.9)	0.922 *
Postpartum age (days), mean (SD)	58 (30.0)	55 (22.1)	0.749 *
Education level *n*, *(%)*			
Incomplete primary education	2 (22.2)	3 (10.0)	0.343
Complete primary education	1 (11.1)	1 (3.3)	
Incomplete secondary education	1 (11.1)	5 (16.7)	
Complete secondary education	3 (33.3)	19 (63.3)	
Complete higher education	2 (22.2)	2 (6.7)	
Family income level *n*, (%) ^a^			
<1 Minimum wage	4 (44.4)	19 (63.3)	0.312
>1 Minimum wage	5 (55.6)	11 (36.7)	
Type of delivery *n*, (%)			
Vaginal	2 (22.2)	11 (36.7)	0.420
Caesarian	7 (77.8)	19 (63.3)	
Parity status *n*, (%)			
Primiparous	6 (66.7)	11 (36.7)	0.111
Multtiparous	3 (33.3)	19 (63.3)	
BMI classification (kg/m^2^), (%) ^b^			
Low weight	0 (0)	0 (0)	0.754
Normal	3 (33.3)	14 (46.7)	
Overweight	4 (44.5)	10 (33.3)	
Obese	2 (22.2)	6 (20.0)	
Type of maternal breastfeeding *n*, (%)			
Exclusive maternal breastfeeding	8 (88.9)	25 (83.3)	0.685
Maternal breast milk and other milks	1 (11.1)	5 (16.7)	
Calory intake (Kcal/day), mean (SD)	2624.6 (453.7)	3464.5 (873.4)	0.001 *
Intake of alpha-tocopherol (mg/day), mean (SD)	6.8 (2.1)	9.3 (3.6)	0.013 *
Intake of total fat (g/dia), mean (SD)	58.5 (12.8)	73.3 (27.8)	0.033 *

*n*: number. BMI: Body Mass Index; SD: Standard deviation; Chi-square test. * *t*-test for independent samples used for the variables maternal age, postpartum age, calorie consumption, alpha-tocopherol and total fat; ^a^ Brazilian minimum wage per month = US$ 291.5; ^b^ WHO classification, 2000. *p*-value = level of significance (*p* < 0.05 = statistically significant).

## References

[1] Victora C.G., Bahl R., Barros A.J.D., França G.V.A., Horton S., Krasevec J., Murch S., Sankar M.J., Walker N., Rollins N.C. (2016). Breastfeeding in the 21st century: Epidemiology, mechanisms, and lifelong effect. Lancet.

[2] Debier C. (2007). Vitamin E during pre- and postnatal periods. Vitamins & Hormones.

[3] Brasil (2015). Saúde da Criança: Aleitamento Materno e Alimentação Complementar.

[4] Traber M.G., Erdman J.W., Macdonald I.A., Zeisel S.H. (2012). Vitamin E. Present Knowledge in Nutrition.

[5] Schulpis K.H., Michalakakou K., Gavrili S., Karikas G.A., Lazaropoulou C., Vlachos G., Bakoula C., Papassotiriou I. (2004). Maternal-neonatal retinol and alpha-tocopherol serum concentrations in Greeks and Albanians. Acta Paediatr..

[6] Fares S., Feki M., Khouaja-Mokrani C., Sethom M.M., Jebnoun S., Kaabachi N. (2015). Nutritional practice effectiveness to achieve adequate plasma vitamin A, E and D during the early postnatal life in Tunisian very low birth weight infants. J. Matern.-Fetal Neonatal Med..

[7] Kositamongkol S., Suthutvoravut U., Chongviriyaphan N., Feungpean B., Nuntnarumit P. (2011). Vitamin A and E status in very low birth weight infants. J. Perinatol..

[8] Rodrigues K.D.S.R. (2016). Estado Nutricional em Vitamina E de Mães e Crianças Pré-Termo e Termo do Nascimento aos 3 Meses Pós-Parto. Ph.D. Thesis.

[9] Lima M.S.R., Dimenstein R., Ribeiro K.D.S. (2014). Vitamin E concentration in human milk and associated factors: A literature review. J. Pediatria.

[10] Hampel D., Shahab-Ferdows S., Islam M.M., Peerson J.M., Allen L.H. (2017). Vitamin Concentrations in Human Milk Vary with Time within Feed, Circadian Rhythm, and Single-Dose Supplementation. J. Nutr..

[11] Debier C., Pottier J., Goffe C., Larondelle Y. (2005). Present knowledge and unexpected behaviours of vitamins A and E in colostrum and milk. Livest. Prod. Sci..

[12] Tijerina-Sáenz A., Innis S., Kitts D. (2009). Antioxidant capacity of human milk and its association with vitamins A and E and fatty acid composition. Acta Paediatr..

[13] Stuetz W., Carrara V., Mc Gready R., Lee S., Sriprawat K., Po B., Hanboonkunupakarn B., Grune T., Biesalski H., Nosten F. (2016). Impact of Food Rations and Supplements on Micronutrient Status by Trimester of Pregnancy: Cross-Sectional Studies in the Maela Refugee Camp in Thailand. Nutrients.

[14] Szlagatys-Sidorkiewicz A., Zagierski M., Jankowska A., Łuczak G., Macur K., Bączek T., Korzon M., Krzykowski G., Martysiak-Żurowska D., Kamińska B. (2012). Longitudinal study of vitamins A, E and lipid oxidative damage in human milk throughout lactation. Early Hum. Dev..

[15] Olafsdottir A.S., Wagner K.-H., Thorsdottir I., Elmadfa I. (2001). Fat-Soluble Vitamins in the Maternal Diet, Influence of Cod Liver Oil Supplementation and Impact of the Maternal Diet on Human Milk Composition. Ann. Nutr. Metab..

[16] Garcia L.R.S., Ribeiro K.D.d.S., de Araújo K.F., Azevedo G.M.M., Pires J.F., Batista S.D., Dimenstein R. (2009). Níveis de alfa-tocoferol no soro e leite materno de puérperas atendidas em maternidade pública de Natal, Rio Grande do Norte. Revista Brasileira de Saúde Materno Infantil.

[17] Clemente H.A., Ramalho H.M.M., Lima M.S.R., Grilo E.C., Dimenstein R. (2015). Maternal Supplementation with Natural or Synthetic Vitamin E and Its Levels in Human Colostrum. J. Pediatr. Gastroenterol. Nutr..

[18] Gaur S., Kuchan M.J., Lai C.-S., Jensen S.K., Sherry C.L. (2017). Supplementation with *RRR-* or *all-rac* -α-Tocopherol Differentially Affects the α-Tocopherol Stereoisomer Profile in the Milk and Plasma of Lactating Women. J. Nutr..

[19] Traber M.G. (2014). Vitamin E Inadequacy in Humans: Causes and Consequences. Adv. Nutr..

[20] Medeiros J.F.P., da Silva Ribeiro K.D., Lima M.S.R., das Neves R.A.M., Lima A.C.P., Dantas R.C.S., da Silva A.B., Dimenstein R. (2016). α-Tocopherol in breast milk of women with preterm delivery after a single postpartum oral dose of vitamin E. Br. J. Nutr..

[21] Lira L.Q. (2017). Efeito de Dois Protocolos de Suplementação Materna com Alfa-Tocoferol Sobre o Soro e o Leite de Lactantes até 60 Dias Pós-Parto. Ph.D. Thesis.

[22] de Melo L.R.M., Clemente H.A., Bezerra D.F., Dantas R.C.S., Ramalho H.M.M., Dimenstein R. (2017). Effect of maternal supplementation with vitamin E on the concentration of α-tocopherol in colostrum. J. Pediatria.

[23] Cortês da Silva A.L., da Silva Ribeiro K.D., Miranda de Melo L.R., Fernandes Bezerra D., Carvalho de Queiroz J.L., Santa Rosa Lima M., Franco Pires J., Soares Bezerra D., Osório M.M., Dimenstein R. (2017). Vitamina e no leite humano e sua relação com o requerimento nutricional do recém-nascido a termo. Revista Paulista de Pediatria.

[24] Ma D., Ning Y., Gao H., Li W., Wang J., Zheng Y., Zhang Y., Wang P. (2014). Nutritional Status of Breast-Fed and Non-Exclusively Breast-Fed Infants from Birth to Age 5 Months in 8 Chinese Cities. Asia Pac. J. Clin. Nutr..

[25] Antonakou A., Chiou A., Andrikopoulos N.K., Bakoula C., Matalas A.-L. (2011). Breast milk tocopherol content during the first six months in exclusively breastfeeding Greek women. Eur. J. Nutr..

[26] GPower Software. http://www.gpower.hhu.de.

[27] Faul F., Erdfelder E., Lang A.-G., Buchner A. (2007). G*Power 3: A flexible statistical power analysis program for the social, behavioral, and biomedical sciences. Behav. Res. Methods.

[28] de Lira L.Q., Lima M.S.R., de Medeiros J.M.S., da Silva I.F., Dimenstein R. (2013). Correlation of vitamin A nutritional status on alpha-tocopherol in the colostrum of lactating women: Relationship of serum retinol and alpha-tocopherol in colostrum. Matern. Child Nutr..

[29] Nierenberg D.W., Nann S.L. (1992). A method for determining concentrations of retinol, tocopherol, and five carotenoids in human plasma and tissue samples. Am. J. Clin. Nutr..

[30] (2000). Dietary Reference Intakes for Vitamin C, Vitamin E, Selenium, and Carotenoids.

[31] Martin S.S., Blaha M.J., Elshazly M.B. (2013). Comparison of a novel method vs. the Friedewald equation for estimating low-density lipoprotein cholesterol levels from the standard lipid profile. JAMA.

[32] Araújo M.O.D., Guerra T.M. (2007). Alimentos per Capita.

[33] Tomita L.Y., Cardoso M.A. (2002). Relação de Medidas Caseiras, Composição Química e Receitas de Alimentos Nipo-Brasileiros.

[34] Virtual Nutri Plus. http:/www.virtualnutriplus.com.br/.

[35] Schweigert F.J., Bathe K., Chen F., Boscher U., Dudenhausen J.W. (2004). Effect of the stage of lactation in humans on carotenoid levels in milk, blood plasma and plasma lipoprotein fractions. Eur. J. Nutr..

[36] Didenco S., Gillingham M.B., Go M.D., Leonard S.W., Traber M.G., McEvoy C.T. (2011). Increased vitamin E intake is associated with higher α-tocopherol concentration in the maternal circulation but higher α-carboxyethyl hydroxychroman concentration in the fetal circulation. Am. J. Clin. Nutr..

[37] Xue Y., Campos-Gimenez E., Redeuil K.M., Leveques A., Actis-Goretta L., Vinyes-Pares G., Zhang Y., Wang P., Thakkar S.K. (2017). Concentrations of Carotenoids and Tocopherols in Breast Milk from Urban Chinese Mothers and Their Associations with Maternal Characteristics: A Cross-Sectional Study. Nutrients.

[38] Dimenstein R., Medeiros A.C.P., Cunha L.R.F., Araújo K.F., Dantas J.C.O., Macedo T.M.S., Stamford T.L.M. (2010). Vitamin E in human serum and colostrum under fasting and postprandial conditions. J. Pediatria.

[39] Jiang J., Xiao H., Wu K., Yu Z., Ren Y., Zhao Y., Li K., Li J., Li D. (2016). Retinol and α-tocopherol in human milk and their relationship with dietary intake during lactation. Food Funct..

[40] Martysiak-Żurowska D., Szlagatys-Sidorkiewicz A., Zagierski M. (2013). Concentrations of alpha- and gamma-tocopherols in human breast milk during the first months of lactation and in infant formulas: Tocopherols in human milk and infant formulas. Matern. Child Nutr..

[41] Leonard S.W., Good C.K., Gugger E., Traber M.G. (2004). Vitamin E bioavailability from fortified breakfast cereal is greater than that from encapsulated supplements. Am. J. Clin. Nutr..

[42] Bruno R.S., Leonard S.W., Park S.I., Zhao Y., Traber M.G. (2006). Human vitamin E requirements assessed with the use of apples fortified with deuteriumlabeled alpha-tocopheryl acetate. Am. J. Clin. Nutr..

[43] Traber M.G., Leonard S.W., Bobe G., Fu X., Saltzman E., Grusak M.A., Estande S.L. (2015). α-Tocopherol Disappearance Rates from Plasma Depend on Lipid Concentrations: Studies Using Deuterium-Labeled Collard Greens in Younger and Older Adults. Am. J. Clin. Nutr..

[44] Jensen S.K., Johannsen A.K.B., Hermansen J.E. (1999). Quantitative secretion and maximal secretion capacity of retinol, β-carotene and α-tocopherol into cows’ milk. J. Dairy Res..

[45] Weiss W.P., Wyatt D.J. (2003). Effect of Dietary Fat and Vitamin E on α-Tocopherol in Milk from Dairy Cows. J. Dairy Sci..

[46] Lauridsen C., Engel H., Jensen S.K., Craig A.M., Traber M.G. (2002). Lactating Sows and Suckling Piglets Preferentially Incorporate RRR- over All-rac-alpha-Tocopherol into Milk Plasma and Tissues. J. Nutr..

[47] Wang Y., Tong J., Li S., Zhang R., Chen L., Wang Y., Zheng M., Wang M., Liu G., Dai Y. (2011). Over-Expression of Human Lipoprotein Lipase in Mouse Mammary Glands Leads to Reduction of Milk Triglyceride and Delayed Growth of Suckling Pups. PLoS ONE.

[48] Monks J., Huey P.U., Hanson L., Eckel R.H., Neville M.C., Gavigan S.A. (2001). lipoprotein-containing particle is transferred from the serum across the mammary epithelium into the milk of lactating mice. J. Lipid Res..

[49] Mardones P., Rigotti A. (2004). Cellular mechanisms of vitamin e uptake: Relevance in α-tocopherol metabolism and potential implications for disease. J. Nutr. Biochem..

